# Epidemiological studies on forestomach disorders in cattle and buffaloes

**DOI:** 10.14202/vetworld.2015.1063-1067

**Published:** 2015-09-15

**Authors:** A. K. Sharma, P. S. Dhaliwal, C. S. Randhawa

**Affiliations:** Department of Veterinary Medicine, Guru Angad Dev Veterinary and Animal Sciences University, Ludhiana, Punjab, India

**Keywords:** abomaso duodenal ulcerations, diaphragmatic hernia, forestomach, incidence, reticulum, rumen, traumatic reticuloperitonitis

## Abstract

**Aim:**

To study epidemiology of forestomach (reticuloruminal, omasal, and abomasal) disorders in cattle and buffaloes.

**Materials and Methods:**

The 106 buffaloes and 32 cattle referred for treatment to the university large animals teaching hospital with the complaint of gastrointestinal diseases constituted the study material. The cases were diagnosed based on history, clinical examination, hematology, biochemistry, radiography, peritoneal fluid analysis and ultrasonography, rumenotomy, and postmortem. A questionnaire was prepared containing important information on housing, husbandry practices, including feeding practices and individual animal information *viz*. age, species, month of the year, parity, gestation (month), and recent parturition. The animals were divided into eight groups and analysis of variance was performed to study risk factors associated with each condition.

**Results:**

The forestomach disorders are widely prevalent in cattle and buffaloes between April and October, during summer and rainy season (90%) and constituted a significant proportion of diseased cows and buffaloes (138/1840) at the hospital. Different forestomach disorders and their prevalence was: Diaphragmatic hernia (DH) 17%, traumatic reticuloperitonitis (TRP) 14%, idiopathic motility disorder or vagus indigestion (VI) 22%, adhesive peritonitis (AP) 13%, frank exudative peritonitis (FEP) 12%, reticular abscess (RA) 8%, ruminal and omasal impaction (RI) 5%, and abomaso duodenal ulceration (ADU) 9%. DH and RA were significantly more common in buffaloes as compared to cattle. Similarly, impactions were more in buffaloes but its incidence was very low (5%). ADU was present in buffalo as commonly as in cows. Exclusive feeding of wheat straw was present in an abysmally low number of animals and hence could not be considered the cause of these disorders. DH was significantly higher in buffaloes (>5 years) of 5-8 years of age and TRP, VI and AP were observed in cattle and buffalo of 2-8 years of age during the second half of gestation to 1 month post-calving. FEP (12%) occurred more commonly within 1 month of parturition.

**Conclusions:**

DH, TRP, frank and AP and ADU are causes of the widely prevalent forestomach (reticuloruminal, omasal and abomasal) disorders in cattle and buffaloes.

## Introduction

Forestomach disorders occupy the center stage of large animal internal medicine and are one of the earliest reported problems of dairy animals. The anatomical structure and location makes them inaccessible to common clinical examination aids and they are placed pretty close to important organs like heart, lungs, liver and spleen. At times their pathologies involve these organs and vice-versa. Since long, these disorders were being referred to on the basis of symptoms, e.g. digestive disorders with overloading of forestomach and accumulation of gas in the dorsal rumen [1]. In the absence of proper etiological understanding, the nomenclature and classifications of these disorders were not properly defined and these were often subjectively referred to as vagus indigestion (VI) syndrome [2], a term coined without clear-cut evidence of major involvement of vagus nerve. In India, most of these disorders are simply referred to as impaction due to common findings of loss or considerably decreased fecal output whereas literature refers to primary ruminal impaction as an extremely rare condition in cattle.

The forestomach including reticulum, rumen, omasum and abomasum are the organs primarily affected with pathology such as traumatic reticuloperitonitis (TRP) [3,4], diaphragmatic hernia (DH), abscesses, adhesions, impactions, dilatations, and motility disturbances [5]. All these disorders are manifested by a pattern of similar or somewhat closely related signs such as anorexia, dullness, recurrent or persistent tympany and abdominal distension, etc. which are common and difficult to differentiate from each other. Furthermore, these disorders cause huge economic loss to farmers in the form of animal mortality and loss of production. The nagnitude of loss to dairy industry can be assessed from the fact that abomasal diseases and resulting peritonitis which are part of these forestomach disorders complex had been responsible for more than 15 percent of all the natural deaths in dairy and beef animals.

The cases of forestomach disorders are on rise and had become a major problem of cattle and buffalo in the state. These referred animals constitute maximum number of cases for any problem at university large animal hospital. Their diagnosis is a daunting task because of enormous variability of clinical signs. Epidemiological study of these disorders is, therefore, important for correct diagnosis, rational treatment, and effective prevention strategies.

## Materials and Methods

### Ethical approval

All clinical cases were examined and treated in Large Animal Clinics, Guru Angad Dev Veterinary and Animal Sciences University (GADVASU), Ludhiana, Punjab, India, as per standard examination and treatment procedure.

### Selection of animals

The study was conducted on 138 animals comprising 106 buffaloes and 32 cattle referred for treatment to Large Animal Clinics, Teaching Veterinary Clinical Service Complex (TVCC), GADVASU, Ludhiana, Punjab for the complaint of gastrointestinal diseases.

### Study design

The history was noted and clinical as well as a special examination of gastrointestinal system including rectal was performed. To confirm the presence of condition the animals were subjected to blood work for routine hematology and peripheral blood film examination, biochemistry panel for inflammatory proteins and liver as well as kidney function tests including plasma fibrinogen, total protein, albumin, globulin, creatinine, and blood urea nitrogen, radiography of reticular area for foreign bodies, peritoneal fluid collection and analysis, ultrasonography of abdomen especially reticulum, perireticular region, omasum and abomasum, rumenotomy (40 animals) as well as postmortem (16 animals) of animals died during course of treatment. On the basis of results of these tests, the animals were divided into eight groups. A questionnaire was prepared containing important information on husbandry practices, including rearing system such as peg tied and stall feeding or grazing/range system and feeding practices with respect to availability of sufficient (30-40 kg daily) green fodder, concentrate (more than 2 kg per day) and type of wheat straw, i.e. whether thresher made or reaper machine made individual animal information such as age, species, month, parity, pregnancy, and lactation.

### Statistical analysis

In order to compare the mean of different variables and see the signs of variations of these epidemiological features in different disease groups, analysis of variance was done.

## Results

The majority of the animals in the present study were kept peg tied (90%), only 8.3% of them reared in semi loose and range (2.17%) system. The indoor feeding practices consisted of feeding seasonal chaffed green fodder and wheat straw as well as concentrate. 85% (116/138) of wheat straw was thresher made whereas in only 15% cases it was reaper machine made. More than 80% of animals had good body condition as per body condition score. Different forestomach disorders and their prevalence was: DH 17%, TRP 14%, idiopathic motility disorder or VI 22%, adhesive peritonitis (AP) 13%, frank exudative peritonitis (FEP) 12%, reticular abscess (RA) 8%, ruminal and omasal impaction 5%, and abomaso duodenal ulceration (ADU) 9%. The epidemiological features related to different conditions is included in Tables-1 and -2.

**Table-1:** Seasonal distribution, species differences and feeding practices in functional forestomach disorders.

Groups	DH (24) (%)	TRP (19) (%)	VI (31) (%)	AP (18) (%)	FEP (16) (%)	RA (11) (%)	Impaction (7) (%)	ADU (12) (%)	Overall (%)
November-March	16.66	5.27	16.13	5.55	18.75	0	0	0	10
April-June	29.16	52.63	32.25	27.78	25	27.27	57.15	25	33.33
July-October	54.16	42.10	51.61	66.67	56.25	72.73	42.85	75	56.52
Prevalence	17.4	13.8	22.5	13	11.6	8.0	5.0	8.7	-
Buffalo	95.83	63.15	74.19	77.77	68.75	100	85.71	45.45	77
Cattle	4.17	36.84	15.81	22.22	31.25	0	14.29	55.55	23
Female	100	94.73	96.77	94.44	100	100	100	100	98
Male	0	5.27	3.23	5.55	0	0	0	0	2
Green fodder	95.83	78.94	93.54	100	87.5	100	42.85	83.33	87.7
Concentrate feeding	62.5	84.21	74.19	72.22	87.5	60	85.71	75	74.45
Reaper wheat straw	12.5	21.06	16.13	16.67	6.25	9.10	28.58	16.67	15.33
Peg tied/semi-loose system	100	100	96.67	94.45	100	100	100	100	97.93
Range system (%)	0	0	3.23	5.55	0	0	0	0	2.17

DH=Diaphragmatic hernia, TRP=Traumatic reticuloperitonitis, VI=Vagus ingestion, AP=Adhesive peritonitis, FEP=Frank exudative peritonitis, RA=Reticular abscess, ADU=Abomasoduodenal ulceration

**Table-2:** Variations in the age, reproductive stage and case presentation of different functional forestomach disorders.

Groups	Age (mean±SD)	Parity								Gestation (months)				Recent parturition (mean±SD)
	
		Heifer (%)	1^st^ (%)	2^nd^ (%)	3^rd^ (%)	4^th^ (%)	5^th^ (%)	>5^th^ (%)	Mean±SD	4-5 (%)	6-8 (%)	>8 (%)	Mean±SD	
DH (24)	7.38±3.01	8.33	25	20.83	16.67	16.67	8.33	4.16	2.50±1.64	12.5	29.17	25	4.96±3.90	0.21±0.42
TRP (19)	6.34±2.99	6.66	40	20	33.33	0	0	0	1.42±1.17	11.76	29.41	17.64	3.79±3.94	0.32±0.48
VI (31)	6.71±3.29	19.23	19.23	26.92	23.07	3.84	7.69	0	1.65±1.52	10	23.33	23.33	4.42±4.49	0.23±0.50
AP (18)	8.05±4.12	0	31.25	6.25	31.25	25	0	6.25	2.79±2.15	11.76	58.82	5.88	4.53±3.52	0.21±0.42
FEP (16)	7.00±2.31	0	23.07	30.76	23.07	23.07	0	0	2.00±1.41	6.25	6.25	0	0.63±1.86	0.44±0.51
RA (11)	5.14±2.25	20	50	20	0	10	0	0	1.18±1.17	18.18	36.36	18.18	5.09±3.67	0.00±0.00
Impaction (7)	11.71±3.30	0	0	0	16.66	33.32	33.32	16.66	2.29±2.87	14.29	71.42	0	5.86±2.85	0.00±0.00
ADU (12)	7.73±4.61	16.67	25	8.33	8.33	16.67	8.33	16.67	2.33±2.55	25	21.67	0	4.40±3.80	0.33±0.82
F ratio	2.902								1.703				2.557	1.168
p value	0.007								0.113				0.017	0.325

DH=Diaphragmatic hernia, TRP=Traumatic reticuloperitonitis, VI=Vagus ingestion, AP=Adhesive peritonitis, FEP=Frank exudative peritonitis, RA=Reticular abscess, SD=Standard deviation

### DH

The prevalence of DH was highest from July to October and more in buffaloes as compared to cattle. Any predisposition of particular sex could not be observed. The incidence of DH decreased gradually with an increase in parity. The highest number of cases was in first (6/24) and least was in 5th (1/24) lactation. The average age of group was 7.38±3.01 years and majority (42%) of animals were in 5-8 years age. These groups differed significantly at 1% level vis-à-vis age of constituent animals. The condition was predominantly prevalent in recently parturated animals or in the second half of gestation.

### TRP (penetrating foreign body)

Principally, cases were reported from April to October. Cattle and buffaloes were equally susceptible to this disorder. The average age of affected animals was 6.34±2.99 years. The maximum number of cases was in the first followed by third (4/19) parity. About one-third (29.41%) of animals were in 6-8 month of gestation and 6 (35.3%) were recently calved and within 1 month of lactation.

### VI

All the cases of VI were presented from April to October. The cattle and buffaloes were equally predisposed to the problem. The incidence in cattle and buffaloes was 16% and 84% whereas their population proportion was 23% and 77%, respectively. Similarly, male and female were affected according to their respective ratio in hospital cases without any preference for a particular sex. The cases were more or less uniformly distributed up to third parity the number of which declined sharply afterward. The mean age of animal in this group was 6.71±3.29 years. The age of various groups differed significantly (p≤0.01). Roughly, an equal number of animals were between pregnancy of 6-8 months, above 8 months, non-pregnant and within 30 days of onset of lactation. The mean gestation period for the group was 4.42±4.49 months (i.e. mid-gestation) and was significantly (p≤0.01) different from other groups.

### AP

All but one case were reported from April to October. Cattle 4 (22%), buffaloes 14 (78%), male 1 (6%) and female 17 (94%) were as per their population proportion in the study and maximum number (five, 31.3%) of cases were present in 1st and 3rd parity. Incidentally, no case in heifer was reported. Age of affected animals in this group was 8.05±4.12 years. More than half of the animals (10/17) were in 6-8 month of gestation. Average gestation stage of the affected group was 4.53±3.52 month. Unlike cases of TRP and RA, the problem was not inversely related to the parity.

### FEP

The majority of cases (56%) were observed during July to October. With little deviation, disease was equally distributed in cattle (31%) and buffaloes (69%) relative to their population. Incidentally, all the animals were female. Maximum number of (75%) animals was in the age group of 5-8 years. The average age of animals comprising this group was 7.0±2.31 years. There were 4 (25%) animals in first, 6 (37.4%) in second and an equal number of 3 (18.7%) in third and fourth parity. Maximum number of animals (88%) were either recently parturated or/and were non-pregnant.

### RA

All the affected animals were buffaloes, which is an important finding in the study. All cases were reported from April to October and in female animals. Maximum number (7/11) of cases was in the middle age group of 5-8 years and in the first lactation. The average age of affected buffaloes was 5.14±2.25 years. The average gestation period of the group was 5.09±3.67 months.

### Forestomach impactions

The incidence was quite low as compared to other forestomach dysfunctions (7/138). All the cases were reported from April to August months. The prevalence rate was more in buffaloes (6/7) compared to cattle (1/7). All the affected animals were females. The mean age of affected animals was 11.71±3.30 years and was significantly higher (p≤0.01) than all other groups. In general, the incidence of impaction increased with lactation number but there was no significant difference with respect to parity. The majority (5/7) of animals were 6-8 months pregnant.

### ADU

Most cases were observed between months of April and October. The cattle and buffalo were equally affected. Abomasal ulceration was observed in animals ranging from 3 to 15 year with mean age of 7.30±4.61 years. Contrary to other forestomach conditions the incidence appeared equally distributed with age. All animals were female and most of the animals were either pregnant or recently calved.

## Discussion

There are a few epidemiological studies to ascertain the incidence of the forestomach disorder in cattle and buffaloes. A survey in Denmark observed more than 16% incidence of a foreign body in 1491 dairy cattle slaughtered, whereas routine surveys in slaughtered dairy animals cited an incidence of <2% [6]. An epidemiological study on 61,124 Ayrshire cattle revealed a TRP lactational incidence risk of 0.6% [7]. This ranked above the common diseases, such as prolapse of uterus and vagina, abortion, udder edema, disorders of abomasums, hypomagnesemia, and rumen acidosis. In India, very few such surveys are available, and some inference can be made from the cases presented to hospitals for these problems. In one study, it was observed that 49.6% of cases had a foreign body on rumenotomy. Another study reported 140 cases of DH in a period of 4 years [8]. Similarly, evaluation of 363 radiographs of animals suffering from forestomach disorders revealed 42% suffering from DH [9]. Recent reports have described a high prevalence of hardware disease in buffaloes [10,11].

Forestomach disorders in the present study were recorded in animals of 3-10 years of age. RA were observed in comparatively younger (heifer and 1st lactation) animals whereas impactions in older animals of above 10 years. The age differences were significant (p≤0.01) and important finding of this study. No such study comparing different forestomach disorders is available, and the findings concurred well with sole studies on TRP and DH [8,12]. Decreased risk of TRP with increasing parity has been reported [7] in one such previous study. As TRP has guarded prognosis and often results in death, the decreased risk with increasing parity and age seems to be a survivorship phenomenon among animals that have not been exposed to the disease at an earlier age. High incidence in the second half of gestation till 1 month of parturition concurred partly with other work [12] and was related with straining during calving similar to the present study. Descending gravid uterus in the second half of gestation starts pushing rumen forward, favoring the foreign bodies to perforate the reticulum. FEP cases were significantly more within a month after calving. Periparturient injuries were a strong possibility in these cases. As there is a physiological increase of peritoneal fluid in periparturient period, it might facilitate spread of any introduced infection leading to diffused generalized peritonitis. Similar prevalence of DH in buffaloes was observed, 50% of animals with DH were within 3 months of parturition; whereas another 25% were above 5 months of gestation [8]. One retrospective study on 263 bovine recorded 50% of animals within 20 days of calving [12].

A comparison of different disorders between cattle and buffalo revealed predisposition of buffaloes to DH, RA, and impaction. The high incidence of DH in buffaloes is well-known and is ascribed to innate weakness of diaphragm, or genetic predisposition of this species [8]. However, high incidence of RA and impaction in buffalo is a new and interesting finding which could not be explained. Higher incidence of TRP in buffalo as compared to cattle in India has been described previously. There is a possibility of different response to the trauma in cattle and buffaloes. There could be less warding off tendency to infection in cows as compared to buffaloes. That is why few reports on RA are available [13] in a cow in spite of good number of studies on the TRP and associated disorders. Nevertheless, the different response of cows and buffaloes could not be proved by this study and needed further substantiation. Likewise, the prevalence of ADU was observed in buffaloes as commonly as in cows. A large number of studies have described the prevalence of ADU in cows [14]. In India, only a few reports on ADU are available, and this work seemed the first organized study on the prevalence of ADU particularly in buffaloes.

## Conclusion

Therefore, we conclude that forestomach disorders are widely prevalent in cattle and buffaloes during summer and rainy season (90%). Among these, DH was substantially more in young buffaloes (5-8 years), in the second half of gestation to 1 month of calving and its risk decreases with increase in parity. TRP, VI, and AP was observed in cattle and buffalo of 2-8 years of age during the second half of gestation to 1 month post-calving. DH, RA and impactions were significantly more in buffaloes, as compared to cattle. The incidence of impaction of different stomach compartments amongst these disorders was low (5%) and significantly more in older (11.71±3.30 years) animals. ADU was present in buffaloes as commonly as in cows.

## Authors’ Contributions

AKS conducted the trial, collected samples, analyzed data, drafted and revised the manuscript. PSD monitored the trial and preparation of the manuscript. CSR provided suggestions to improve the trial procedure and manuscript.

## References

[1] Rosenberger G., Dirksen G., Grunder H.D., Grunert E., Krause D., Atober H. (1979). Clinical Examination of Cattle.

[2] Rebhun W.C., Fubini S.L., Timothy T.K. (1988). Vagus indigestion in cattle: clinical feature, causes, treatment and long term follow up of 118 cases. Compend. Contin. Edu. Pract. Vet.

[3] Chanie M., Tesfaye D. (2012). Clinico-pathological findings of metallic and non-metallic foreign bodies in dairy cattle: A review. Acad. J. Anim. Dis.

[4] Ghanem M.M. (2010). A comparative study on traumatic reticuloperitonitis and traumatic pericarditis in Egyptian cattle. Turk. J. Vet. Anim. Sci.

[5] Watts A.S., Tulley W.J. (2013). Case report: Sequele of traumatic reticuloperitonitis in a Friesian dairy cow. N. Z. Vet. J.

[6] Cramers T., Mikkelsen K.B., Andersen P., Enevoldsen C., Jensen H.E. (2005). New types of foreign bodies and the effect of magents in traumatic reticulitis in cows. Vet. Rec.

[7] Grohn Y.T., Bruss M.L. (1990). Effect of disease, production and season on traumatic reticuloperitonitis and ruminal acidosis in cattle. J. Dairy Sci.

[8] Krishnamurthy D., Nigam J.M., Deshpande K.S., Peshin P.K., Singh S.C., Sharma D.N. (1983). Bovine diaphragmatic hernia: An analysis of 140 clinical cases. Indian Vet. J.

[9] Nigam J.M., Singh A.P., Mirakhur K.K. (1988). Radiographic diagnosis of bovine thoracic disorders. Mod. Vet. Pract.

[10] Al-Abadi O.S., Abu-Seida A.M., Al-Hussainy S.M. (2014). Studies on rumen magnet usage to prevent hardware disease in buffaloes. Vet. World.

[11] Aref N.M., Abdel-Hakiem M.A.H. (2013). Clinical and diagnostic methods for evaluation of sharp foreign body syndrome in buffaloes. Vet. World.

[12] Singh M. (2002). Evaluation of Surgically Treated Gastrointestinal Disorders in Bovines. M.V. Sc. Thesis.

[13] Fubini S.L., Normand G., Ducharme G.N., Hollis N., Erb N.H., Donald F.S., Williams C., Rebhun W.C. (1989). Failure of omasal transport attributable to perireticular abscess formation in cattle, 29 cases (1980-1986). J. Am. Vet. Med. Assoc.

[14] Hund A., Dzieciol M., Schmitz-Esser S., Wittek T. (2015). Characterization of mucosa-associated bacterial communities in abomasal ulcers by pyrosequencing. Vet. Microbiol.

